# Conductance-Aware Quantization Based on Minimum Error Substitution for Non-Linear-Conductance-State Tolerance in Neural Computing Systems

**DOI:** 10.3390/mi13050667

**Published:** 2022-04-24

**Authors:** Chenglong Huang, Nuo Xu, Wenqing Wang, Yihong Hu, Liang Fang

**Affiliations:** 1 Institute for Quantum Information & State Key Laboratory of High Performance Computing, National University of Defense Technology, Changsha 410073, China; huangchenglong16@nudt.edu.cn (C.H.); wangwenqing@nudt.edu.cn (W.W.); huyihong137@nudt.edu.cn (Y.H.); lfang@nudt.edu.cn (L.F.); 2 College of Computer, National University of Defense Technology, Changsha 410073, China; 3 Wuhan National Laboratory for Optoelectronics, Wuhan 430074, China

**Keywords:** ReRAM, non-linear conductance levels, conductance-aware quantization

## Abstract

Emerging resistive random-access memory (ReRAM) has demonstrated great potential in the achievement of the in-memory computing paradigm to overcome the well-known “memory wall” in current von Neumann architecture. The ReRAM crossbar array (RCA) is a promising circuit structure to accelerate the vital multiplication-and-accumulation (MAC) operations in deep neural networks (DNN). However, due to the nonlinear distribution of conductance levels in ReRAM, a large deviation exists in the mapping process when the trained weights that are quantized by linear relationships are directly mapped to the nonlinear conductance values from the realistic ReRAM device. This deviation degrades the inference accuracy of the RCA-based DNN. In this paper, we propose a minimum error substitution based on a conductance-aware quantization method to eliminate the deviation in the mapping process from the weights to the actual conductance values. The method is suitable for multiple ReRAM devices with different non-linear conductance distribution and is also immune to the device variation. The simulation results on LeNet5, AlexNet and VGG16 demonstrate that this method can vastly rescue the accuracy degradation from the non-linear resistance distribution of ReRAM devices compared to the linear quantization method.

## 1. Introduction

Recently, deep neural networks (DNN) have been the core of the artificial intelligence applications such as image classification [1,2], object detection [3] and speech recognition [4,5], and so on. However, it is not easy to bring the power and latency consumption down when a DNN is directly established in a conventional computer in which a large amount of data need to be carried and processed among separate processing (CPUs and GPUs) and data storage components such as the cache, DRAM, disks, etc., due to the bottleneck of the conventional von-neumann architecture processors; this is called the “memory wall” in the memory domain. Therefore, an increasing number of research works focus on achieving DNN in in-memory computing paradigms to overcome the “memory wall” problem in von-neumann architecture [6,7,8,9,10]. Among them, the emerging non-volatile memory device, especially the resistive random-access memory (ReRAM) [11], has been considered as a promising candidate as the basic unit for in-memory computing due to its high density, fast switch speed and low power consumption. Owning to its physical properties and crossbar structure, the ReRAM based crossbar array (RCA) can accelerate and dominate multiplication-and-accumulation (MAC) operations in DNN that can efficiently reduce the computation complexity from $O(n^{2})$ to O(1). Furthermore, ReRAM can have multiple conductance values by material engineering, which is attractive for DNN applications, as a weight representation [12,13].

Previous research works have demonstrated that multi-valued ReRAM devices cannot be tuned into an arbitrary conductance state due to the conductive mechanism of ReRAM [13,14,15,16,17,18]. In most cases, the multi-valued ReRAM demonstrates the discrete conductance states with limited numbers. Thus, weights trained in the software should be quantized before mapping to the discrete conductance states. Some research works chose the linear weights’ criterion to quantize the weights based on the assumption that the conductance states provided by the ReRAM device are linear in distribution [8,19,20]. However, the actual experimental results on multi-valued ReRAM demonstrate that the distribution of the conductance values are non-linear [21,22]. Therefore, quantizing weights based on a linear criterion will conduct a large deviation when mapping the quantized weights to the non-linear conductor states, which lowers the inference accuracy of RCA-based DNN.

Some research works have made efforts to address the adverse effects of the distribution of the non-linear conductance level of ReRAM devices. The methods can be roughly divided into two categories. The first category is to directly map the linear distribution weight criteria to the selected conduct state criteria with the linear distribution obtained from the ReRAM. In this way, the accuracy rate would not experience loss after mapping. However, it requires that the ReRAM has a large number of conduct states in order to provide enough conductance state criteria with a linear distribution. Otherwise, the large computing deviation should be generated after mapping, as the conductance state criteria with a non-linear distribution have to be mapped to the linear distribution weight criteria from the DNN, or the number of the weight criteria should be reduced to match the linear conductance state criteria. To further reduce the possibility of accuracy loss after mapping, the reverse mapping methods regarding the weight pre-processing are proposed as the second category of dealing with the non-linear problem. The core idea is to align the weight criteria that will be used for training in DNN with the actual conductance state criteria of ReRAM. In this research field, the align or quantization strategies are critical for reducing the loss of the accuracy rate. For example, Dongseok et al. proposed an adaptive quantization method that can easily transfer the weights into real synaptic devices with high performance [23]. In their method, the weights were quantized in the training process based on the measured conductance behavior, and then they obtained accuracy near to the baseline on a fully connected neural network (784×256×10). However, their method was evaluated with a shallow network, and was not tested on a deeper network such as AlexNet and VGG16. Jilan Lin et al. proposed a resistance-aware quantization method that rescues the accuracy degradation caused by three types of non-linear resistance distribution of RRAM devices [21,22]. First, the quantized weight criteria were determined by the actual conductance states of ReRAM. Then, they proposed three weight-boundary decision functions for three types of resistance distribution. The weights in DNN that fall within the decision boundary are represented by the corresponding quantized weight criteria. Their method achieved high accuracy on deeper neural networks such as VGG16. However, the boundary decision functions are only efficient for the specific conductance level distribution of the ReRAM devices they proposed, which lacks generality.

In this paper, a universal and efficient conductance-aware quantization method of weights is proposed to rescue the accuracy degradation of the ReRAM-based DNN accelerator in the limited number of conductance states with a non-linear distribution. The method is suitable for the different conductance level distribution of ReRAM devices. The main contributions of this paper are summarized as follows:

- We observe that the non-linear conductance levels can result in more conductance representations than linear conductance levels when a pair of differential ReRAM devices are employed to map a weight, which effectively holds the inference accuracy in software after the weights are quantized.
- The weight quantization criteria are generated based on the non-linear conductance values of a pair of differential ReRAM devices. The method of minimum error substitute (MES) is employed in the quantization process to determine the quantized weight locations in the software network, which provides a universal quantization method with a different conductance value distribution.
- The proposed MES-based conductance-aware quantization is evaluated with LeNet, AlexNet and VGG16, including the consideration of the device variation.

Our paper is organized as follows. Section 2 briefly introduces the preliminaries of ReRAM and RCA-based DNN. Section 3 presents the MES-based conductance-aware quantization method. Section 4 demonstrates and discusses the simulation results of employing our quantization method in different neural networks achieved in RCA. Different non-linear conductance distributions and device variations are also considered in this simulation. Section 5 concludes this paper.

## 2. Preliminary

### 2.1. The Characteristics of the ReRAM

**Figure 1:**
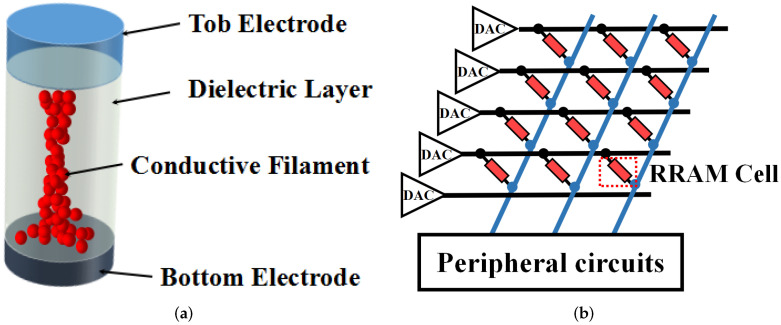
(**a**) Device structure of the ReRAM cell. (**b**) The structure of an ReRAM crossbar array.

Resistive random-access memory (ReRAM) is one of the emerging non-volatile devices that is also regarded as a kind of memristor [11,24]. Figure 1a shows the typical device structure of a ReRAM device, which consists of a stack of metallic top electrodes, an insulating metal-oxide layer and a metallic bottom electrode [11,25,26]. The conductivity of the conductive filament can be tuned by applying the programming voltage across the top electrode and bottom electrode, which alters the conductance of the device. When the programming voltage is applied to the electrodes of the ReRAM cell, the conductive filament grows from one electrode towards the other and eventually connects the two electrodes together, which increases the conductance of the ReRAM cell. Applying this voltage in reverse, the filament will break up and melt, corresponding to a decrease in the conductance of the ReRAM cell. For most of the ReRAM devices, the growing up and melting of the conductive filament can be regarded as a transient process. In other words, the device will be switched between two conductance states of high conductance state (HCS) and low conductance state (LCS). With the help of material engineering, some devices exhibit obvious multiple-value characteristics, for which the length of the conductive filament can be gradually tuned by different amplitudes or times of the programming voltage to achieve multiple conductance states between the two extreme values. This kind of ReRAM device is called a multi-value ReRAM, which is attractive in DNN applications due to its stronger ability of representing weights, versus a binary-value ReRAM cell.

### 2.2. Reram-Based DNN

#### 2.2.1. Rca-Based MAC Operation

ReRAM cells can be easily integrated into a circuit structure of a crossbar array as it is shown in Figure 1b. The ReRAM-based crossbar array (RCA) is also a friendly natural platform to achieve a DNN arithmetic with high parallelism and area efficiency. The original computation of the convolution and fully connected layer in DNN is based on the weighted summation with input feature maps that can be implemented in the RCA in an in-memory computing fashion, as shown in Figure 1b. In ReRAM-based DNN, the input data are encoded as digital signals and further converted into analog voltages by the digital-to-analog converters (DAC), which are applied to the rows of the RCA. The output current in each column reflects the result of the multiplication and accumulation (MAC) between the input data and weights. Output current signals will be further processed by peripheral circuits in order to transmit the results to the next RCA. The detailed computing mechanism of MAC in RCA is demonstrated, as follows.

A DNN with the weights of n×m can be stored in an n×m RCA, and the activations can be converted to voltages. Then, the MAC operation can be accomplished in RCA according to the Ohm’s law and Kirchhoff’s law, which can be expressed as:

(1) $$I_{out[m]}=\sum_{i=1}^{n}V_{i}\times G_{im}$$

where $I_{out[m]}$ is the column output current of the ReRAM crossbar, $G_{im}$ is the conductance of the ReRAM cell and $V_{i}$ is the input voltage of the ReRAM crossbar. Based on the RCA, the MAC operation in DNN can be carried out in an in-memory computing manner, which means the RCA can be intensively employed to accelerate MAC operations in DNN [8,9,10].

#### 2.2.2. Accelerating the MAC Operations in DNN Based on RCA Hardware

To achieve the MAC operations of the convolution and fully connected layer in DNN based on RCA, the weights and activations in DNN should be represented by the conductance of the ReRAM cells and the voltages of the rows of the RCA. In order to make the results of the MAC be achieved by the RCA equivalent to the software simulation, the conductance values of the RCA should be equally scaled to the weights of the DNN. However, the conductance of the ReRAM cell is a positive and discrete quantity, which is difficult to isometrically map to the approximate analog weight from the training process of the software. Therefore, several strategies are needed to finish the mapping between the conductance of the ReRAM cell and the weight of the DNN.

(1) Extend the sign and range of the conductance of ReRAM

**Figure 2:**
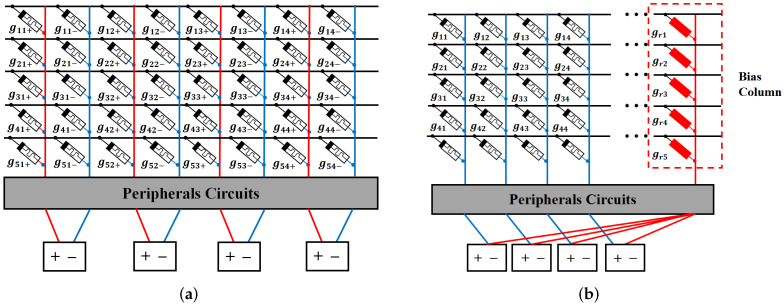
(**a**) The differential pair structure of RCA; (**b**) RCA with a constant bias column.

The conductance of ReRAM is positive, which could not represent the signed weight. In order to represent the non-positive value of the weight, RCA is typically organized as a differential pair, or a constant bias column is added to generate the non-positive values [27,28], as shown in Figure 2. In this way, each weight of DNN is represented by the differential of the two conductance values. In our work, the differential pair strategy is employed to provide the representations for the signed weights. In the following, the word “conductance” represents the differential of the two conductances of ReRAM, unless otherwise stated.

(2) Quantization of the weights in DNN

**Figure 3:**
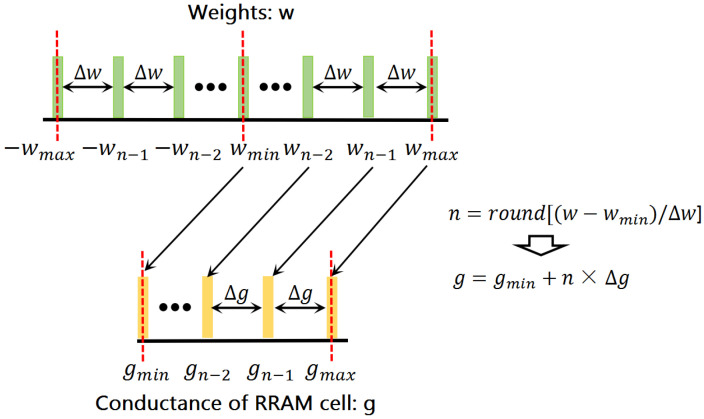
Mapping relationship between conductance levels and quantization levels.

In general, the multi-valued ReRAM can only provide a limited amount of conductance. Therefore, the weights in DNN should be quantized as low-precision representations with the same amount of the conductance to map. In some research works [8,19,20], the weights are typically quantized in a linear manner based on the assumption that the multiple conductance levels in ReRAM devices are linearly distributed. Figure 3 shows the relationship between the conductance levels of ReRAM cells and the linear quantization weight levels in these works. In their assumption, the weights and conductance values of ReRAM are based on the same linear distribution. The relationship between weights in DNN and conductance values of ReRAM in [29,30,31] is shown as:

(2) $$g_{im}=\frac{g_{max}-g_{min}}{w_{max}-w_{min}}\left(w_{im}-w_{min}\right)+g_{min}$$

**Figure 4:**
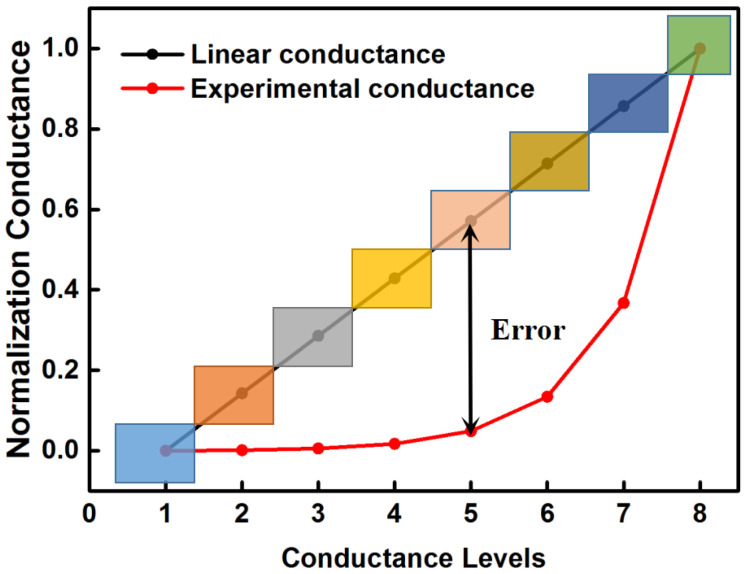
Conductance states distribution of ideal ReRAM cell and actual ReRAM cell.

However, it is hard to obtain the expected linear distribution of the conductance values in a real ReRAM cell. In most of the cases, the programming conductance states in ReRAM shows a non-linear distribution. Therefore, directly mapping the linearly quantized weights to the non-linear conductance levels in the ReRAM cells will result in a high loss in accuracy. Figure 4 shows the deviations between the normalized expected conductance states deduced from the linearly quantized weights based on Equation (2) and the normalized actual conductance states obtained from the real ReRAM device [15]. Such large deviations will cause the actual output of RCA to deviate from the ideal output of software computation and eventually degrade the inference accuracy of the RCA-based DNN.

(3) Strategies of the conductance-aware quantization of weights

Directly mapping the linearly quantized weights to the non-linearly conductance levels results in a large deviation. Thus, the deviation would be eliminated if the quantized weights has the same distribution to the conductance values obtained from the ReRAM cells. Based on this idea, the researchers have proposed some strategies to achieve the conductance-aware quantization of weights. For example, Jilan et al. proposed a conductance-aware quantization method for rescuing the accuracy loss of DNN from the non-linear conductance distribution [22]. They formulated three different non-linear conductance distribution models and then calculated the corresponding decision boundaries. The decision boundaries decide what a particular weight value should be quantized. However, the decision boundaries are calculated based on three different non-linear conductance distribution models that may not be suitable for the new conductance distribution model of ReRAM, which lacks generality.

Therefore, in this paper, we propose a universal conductance-aware quantization method based on minimum error substitution to eliminate the deviation (MES-CAQ) in the mapping process.

## 3. Proposed Method

### 3.1. The Characteristic of the Differential Pair ReRAMs with Non-Linear Distribution of Conductance

**Figure 5:**
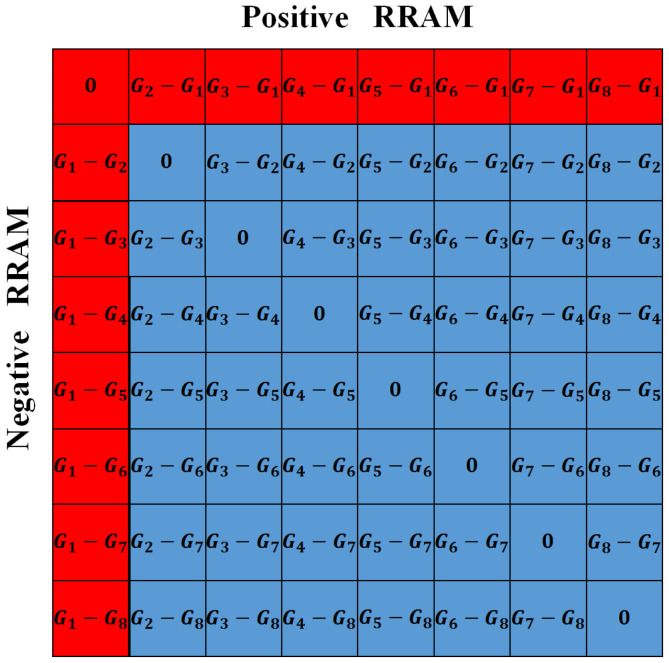
The conductance representations in a differential pair of ReRAM cells.

As it is mentioned in Section 2, the differential pair strategy is used in this work to represent the signed weights. In this part, we will demonstrate that the reason to choose the differential pair strategy is not only to extend the sign of the conductance but also to provide the additional conductance values that may be good to hold the inference accuracy of software. Figure 5 shows the conductance representations provided by a differential pair of ReRAM cells. For the RCA constituted by the ReRAMs with a linear conductance distribution, a differential pair of ReRAM cells can only provide conductance representations with values marked by a red box. In other words, the values in each diagonal are equal. However, if the conductance states of the ReRAM accord with the non-linear distribution, the values in the matrix of Figure 5 are all different, except the diagonal in the middle. Therefore, a differential pair of ReRAM cells can provide more conductance representations for the RCA constituted by the ReRAMs with a non-linear conductance distribution.

### 3.2. Conductance-Aware Quantization Based on Minimum Error Substitution

**Figure 6:**
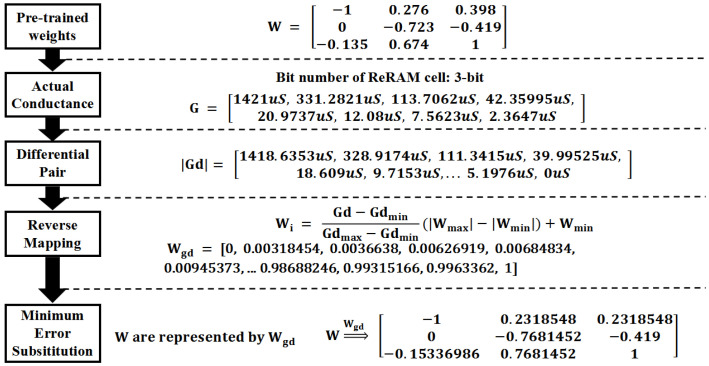
An example of MES-CAQ.

This part is mainly to introduce the MES-CAQ method, which could eliminate the deviation in the mapping process. The whole process is presented in Algorithm 1. We provide a simple example to illustrate the process of MES-CAQ, as shown in Figure 6. The detailed descriptions are as follows. First, the weights **W** can be obtained from a pre-trained DNN model. For a clear demonstration, the pre-trained weights **W** are set as a M×N matrix, as in Equation (3). These weight values are obtained through the software training process. The MES-CAQ method will quantize them into some low precision values with the same number and distribution of the conductance values obtained from the ReRAM cell.

(3) $$\left[\begin{array}{ccc} w_{0,0} & \cdots & w_{0,N-1}\\ \vdots & \ddots & \vdots \\ w_{M-1,0} & \cdots & w_{M-1,N-1} \end{array}\right]$$

**Algorithm 1:** MES-CAQ.
 **Input**:Weights of each layer in pre-trained DNN model W∈M×N; the number of layers of DNN *L*; conductance of 3-bit ReRAM cell $G={\left[g_{1},g_{2},g_{3},g_{4},g_{5},g_{6},g_{7},g_{8}\right]}^{T}$; the mapping relationship between conductance and weights as shown in Equation (4) denoted as F; **Output**:Conductance-aware quantized weights $W_{Q}$;
Conductance representations based on a differential pair of ReRAM cells Gd:$\mathrm{Gd}={\left[g2-g1,g3-g1,g3-g2,...,g8-g6,g8-g7\right]}^{T}=\left[\Delta g1,\Delta g2,\ldots ,\Delta g57\right]$The quantization weight representations based on the differential pair of ReRAM cells $W_{gd}$:$W_{gd}=F\left(\mathrm{Gd}\right)$

$error=\sum_{i=0}^{M-1}\sum_{i=0}^{N-1}{\left(W_{gd}-W_{i,j}\right)}^{2}$



Indices=argmin(error)



$W_{Q}⇐F\left(G_{diff}\right)\left[\mathrm{Indices}\right]$




To finish this quantization, a criterion should first be obtained from the non-linear conductance values of a ReRAM cell. Here, eight non-linear conductance states are assumed to exist in a ReRAM cell and expressed as $G={\left[g1,g2,g3,g4,g5,g6,g7,g8\right]}^{T}$. Based on these disperse conductance values, the conductance values provided by a differential pair of ReRAM cells can be expressed as $\mathrm{Gd}={\left[g2-g1,g3-g1,g3-g2,...,g8-g6,g8-g7\right]}^{T}=\left[\Delta g1,\Delta g2,\ldots ,\Delta g57\right]$. The conductance values in Gd are the real conductance values that will be used in inference computation based on RCA. Therefore, the criterion of the weight quantization can be deduced from Gd based on Equation (4).

(4) $$w_{i}=\frac{\Delta g_{i}-\Delta g_{min}}{\Delta g_{max}-\Delta g_{min}}\left(w_{max}-w_{min}\right)+w_{min}$$

Here, $\Delta g_{i}$ means the conductance representations provided by a differential pair of ReRAM cells, $\Delta g_{min}$ means the minimum value of Gd, $\Delta g_{max}$ means the maximum value of Gd, $w_{max}$ and $w_{min}$ means the maximum value and minimum value of the weights in each layer in the pre-trained DNN and $w_{i}$ means the weights correspond to $g_{i}$. These criterion values are recorded as $W_{gd}$ which can be regarded as a K×1 (K = 57 when G has eight conductance values) matrix and expressed as:

(5) $$W_{gd}=\left[\begin{array}{c} w_{gd_{0}}\\ \vdots \\ w_{gd_{K-1}} \end{array}\right]$$

These criterion values follow the same non-linear distribution with conductance values Gd. The weights of the DNN could be quantized based on these criterion values through a method of minimum error substitute. The detailed process is as follows.

First, calculating the error between each element of W and $W_{gd}$. The results are shown as:

(6) $$error=\left[\begin{array}{ccc} {\left[\begin{array}{c} {\left(w_{gd_{0}}-w_{0,0}\right)}^{2}\\ \vdots \\ {\left(w_{gd_{K-1}}-w_{0,0}\right)}^{2} \end{array}\right]}_{0,0} & \cdots & {\left[\begin{array}{c} {\left(w_{gd_{0}}-w_{0,N-1}\right)}^{2}\\ \vdots \\ {\left(w_{gd_{K-1}}-w_{0,N-1}\right)}^{2} \end{array}\right]}_{0,N-1}\\ \vdots & \ddots & \vdots \\ {\left[\begin{array}{c} {\left(w_{gd_{0}}-w_{M-1,0}\right)}^{2}\\ \vdots \\ {\left(w_{gd_{K-1}}-w_{M-1,0}\right)}^{2} \end{array}\right]}_{M-1,0} & \cdots & {\left[\begin{array}{c} {\left(w_{gd_{0}}-w_{M-1,N-1}\right)}^{2}\\ \vdots \\ {\left(w_{gd_{K-1}}-w_{M-1,N-1}\right)}^{2} \end{array}\right]}_{M-1,N-1} \end{array}\right]$$

The error matrix shown in Equation (6) consists of M×N sub-matrices with a size of K×1. Then, the indices of minimum error in each sub-matrix in Equation (6) are obtained as:

(7) $$Indices=\left[\begin{array}{ccc} argmin({\left[\begin{array}{c} {\left(w_{gd_{0}}-w_{0,0}\right)}^{2}\\ \vdots \\ {\left(w_{gd_{K-1}}-w_{0,0}\right)}^{2} \end{array}\right]}_{0,0}) & \cdots & argmin({\left[\begin{array}{c} {\left(w_{gd_{0}}-w_{0,N-1}\right)}^{2}\\ \vdots \\ {\left(w_{gd_{K-1}}-w_{0,N-1}\right)}^{2} \end{array}\right]}_{0,N-1})\\ \vdots & \ddots & \vdots \\ argmin({\left[\begin{array}{c} {\left(w_{gd_{0}}-w_{M-1,0}\right)}^{2}\\ \vdots \\ {\left(w_{gd_{K-1}}-w_{M-1,0}\right)}^{2} \end{array}\right]}_{M-1,0}) & \cdots & argmin({\left[\begin{array}{c} {\left(w_{gd_{0}}-w_{M-1,N-1}\right)}^{2}\\ \vdots \\ {\left(w_{gd_{K-1}}-w_{M-1,N-1}\right)}^{2} \end{array}\right]}_{M-1,N-1}) \end{array}\right]$$

The range of each element of the indices is from 0 to K − 1, and the size of indices is M×N.

Finally, the elements in **W** are replaced with the corresponding elements of $W_{gd}$ based on the indices obtained in Equation (7). The weight matrix after the quantization can be expressed as:

(8) $$\left[\begin{array}{ccc} W_{gd}\left[Indices_{0,0}\right] & \cdots & W_{gd}[Indices_{0,N-1}\\ \vdots & \ddots & \vdots \\ W_{gd}[Indices_{M-1,0} & \cdots & W_{gd}[Indices_{M-1,N-1} \end{array}\right]$$

These quantized weights can align to the real conductance values without deviation.

## 4. Simulation and Results

### 4.1. Reram Non-Linear Conductance Models and Fitting Functions

**Figure 7:**
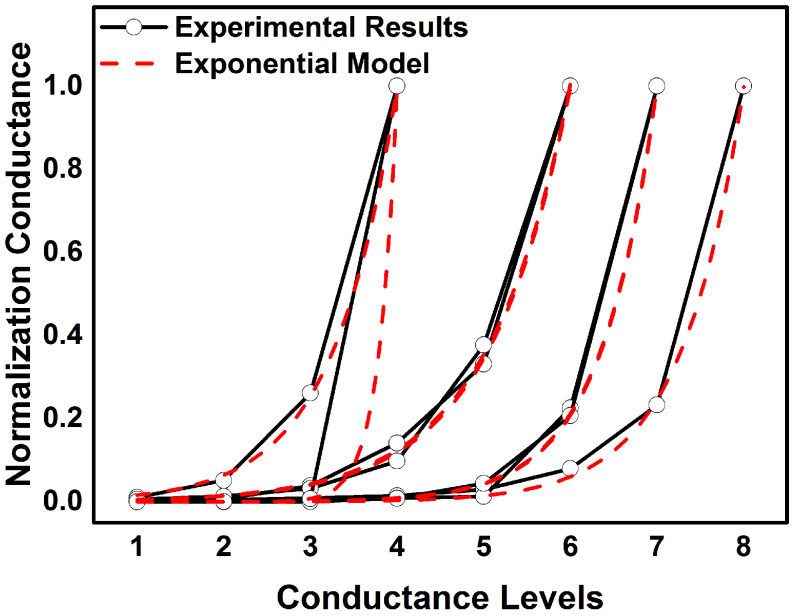
Fitting results for e-exponential model.

To verify the universality of the proposed MES-CAQ, the conductance values of the ReRAM cells in our simulation are extracted from the different conductance models fitted from different ReRAM devices. We use two kinds of conductance distribution models for the simulations. One of them is fitted from the experimental results from several research works on practically multi-valued ReRAM [13,14,15,16,17,18], as shown in Figure 7. These experimental results demonstrate that the normalized conductance values in a ReRAM device are exponentially increased with the conductance levels. Therefore, we use the e-exponential model to fit them as Equation (9).

(9) $$g_{k}=A\times e^{sk}$$

where *A*, *s* are the model parameters, and $g_{k}$ is the k−th level of conductance.

The other models are the non-linear conductance models reported in the previous research works [21,22], set as Equation (10):

(10) $$\left\{\begin{array}{c} g_{k}=C\times K+\delta_{k},DeviatedLinearModel\\ g_{k}=C\times K^{a},Powermodel\\ g_{k}=C\times a^{k},ExponentialModel \end{array}\right.$$

where *a* and *C* are the model parameters, $\delta_{k}$ is the deviation from linear model and $g_{k}$ is the *k*-th level of conductance. The partial parameters in Equation (10) are shown in Table 1.

In the following evaluation process, the non-linear conductance models in Equations (9) and (10) are all considered in a simulation.

### 4.2. The Simulation Results of the MES-CAQ

**Figure 8:**
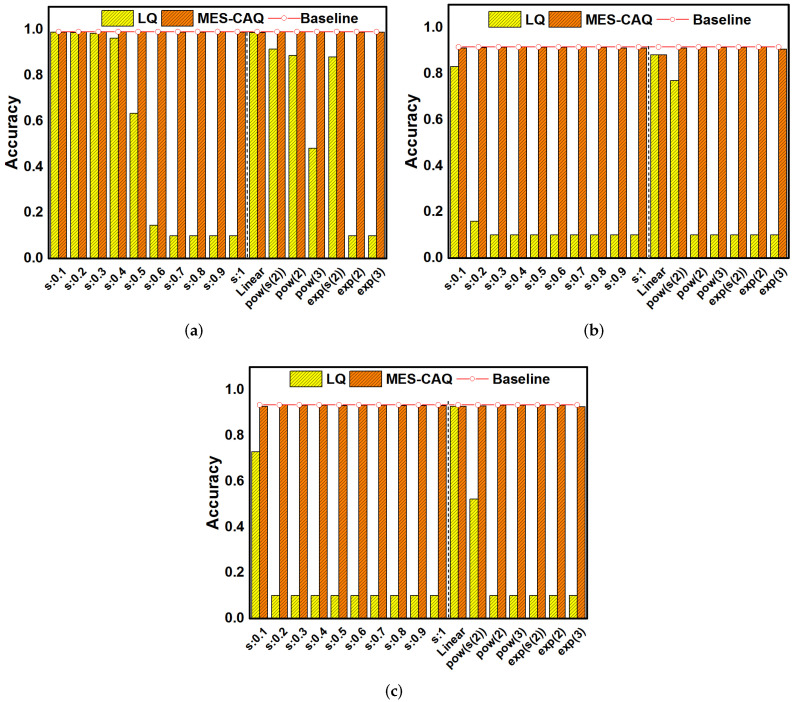
The accuracy results of MES-CAQ in comparison to LQ on (**a**) LeNet5; (**b**) AlexNet; (**c**) VGG16.

In this section, LeNet5 on MNIST datasets, AlexNet on CIFAR-10 datasets and VGG16 on CIFAR-10 datasets are investigated to evaluate the proposed MES-CAQ method. In our evaluation, the bit number of ReRAM devices is set to 3. The *s* in the e-exponential is set from 0.1 to 1 to simulate different degrees of non-linear conductance models that are denoted as “s:0.1∼s:1”. The deviated linear model, power model and exponential model referred in [21,22] are denoted as “Linear”, “pow” and “exp”, respectively. The baseline accuracy of LeNet5, AlexNet and VGG16 is 99.14%, 93.49% and 91.65%. In Figure 8a, using the LQ method to quantize LeNet5 can obtain an inference accuracy close to the baseline when s=0.1∼0.3. The conductance distribution model is approximately linear with a smaller value of *s*. Thus, the deviation between the conductance value and the linearly quantized weight is smaller and the inference accuracy loss is smaller. In the same way, LQ can obtain high inference accuracy when the conductance distribution models are nearly linear, such as ‘Linear’ or power, and the exponential distribution model has a smaller value of a, as shown in Figure 8a. However, LQ will lead to a gradual decrease in the inference accuracy of LeNet5 with the nonlinear degree of the device conductance distribution gradually increasing as a result of s=0.4∼1 and a large value of power and exponential distribution, as shown in Figure 8a. This is due to the large deviation between the linearly quantized weights and non-linear conductance values. A similar regulation is observed in Figure 8b,c. The LQ method can still obtain a high inference accuracy on more complex neural networks and datasets when the conductance distribution is nearly linear. However, when the nonlinearity of the conductance distribution model is further increased, using LQ to quantize the AlexNet and VGG16 will cause a serious loss of inference accuracy. On the contrary, the MES-CAQ-based results always demonstrate no significant degradation in the inference accuracy of DNN. In summary, MES-CAQ can alleviate the inference accuracy loss of the neural network caused by a non-linear conductance distribution.

For a more realistic consideration, the device variation is introduced into the simulation. Based on the previous experimental results on ReRAM devices, the device variation follows a log-normal distribution [32,33,34]. The conductance of the ReRAM devices with device variation can be expressed as

(11) $$g_{nm}=g_{nm}*e^{\theta }$$

where θ∼N(0,σ).

**Figure 9:**
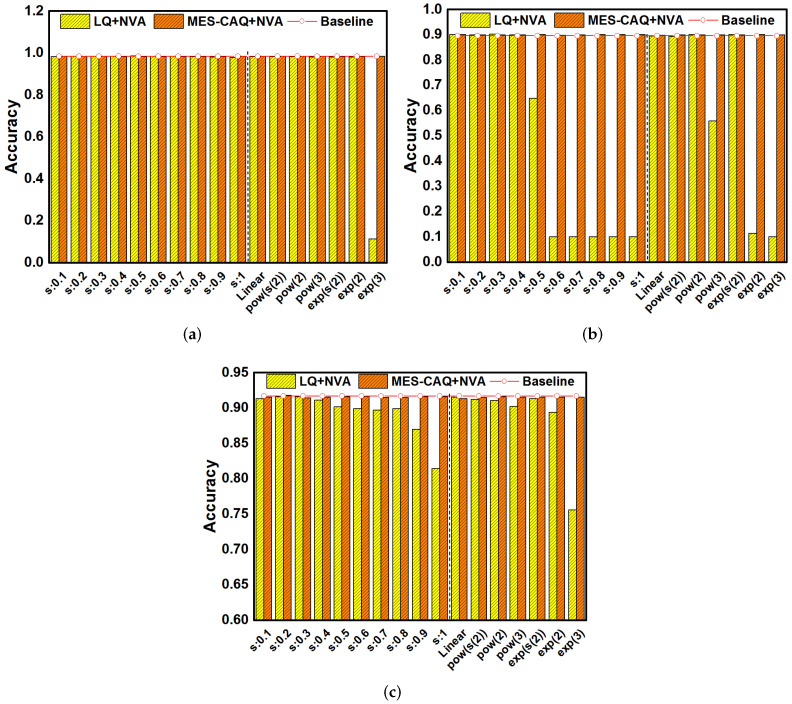
The accuracy results of MES-CAQ in comparison to LQ with device variation on (**a**) LeNet5; (**b**) AlexNet; (**c**) VGG16.

To maintain the accuracy of DNN, we retrain the DNN with MES-CAQ and device variation. For comparison, the DNN with LQ and device variation is also retrained. The variation factor θ is set to 0.5 in simulation. Figure 9a shows that the LQ method can obtain high inference accuracy in most of the non-linear conductance distribution model after retraining with device variation and quantization. However, in the exp(3) model, the inference accuracy of LeNet5 is seriously degraded by the LQ method. In Figure 9b,c, the LQ method can obtain a high inference accuracy when the conductance model is nearly linear. However, there is a trend of decreasing accuracy when the degree of nonlinearity of the conductance distribution model gradually increases. In summary, although the retrain process slows down the degradation of accuracy of the LQ method to some extent, there is still a relatively large gap in accuracy in comparison to the baseline when the nonlinearity of the conductance distribution is high. However, MES-CAQ can always rescue the accuracy of ReRAM-based DNN. The results indicate that the MES-CAQ is an efficient quantization method that is more suitable than LQ in the field of ReRAM-based DNN applications with a non-linear conductance level distribution.

## 5. Conclusions

In this paper, a minimum-error-substitution-based conductance-aware quantization method (MES-CAQ) is proposed to align the weights trained in software-based DNN to the actual conductance values of ReRAM devices in the RCA-based DNN. The pairs of differential ReRAM devices can provide more conductance representations with a non-linear conductance level distribution than with a linear distribution, which can provide more criteria of quantization. The minimum error is employed to determine the quantized weights for weights in DNN. The simulation results demonstrate that our method has almost no loss in accuracy on LeNet5, AlexNet, and VGG16 with different conductance distribution models. In addition, the further simulation results demonstrate that MES-CAQ always performs better than LQ with a non-linear conductance level distribution and device-variation considerations.

## Figures and Tables

**Table 1:** Non-linear model parameters [21,22].

Parameters	$\delta_{k}$	β	*a*
Values	0.10	$\sqrt{2}$, 2, 3	$\sqrt{2}$, 2, 3

## Data Availability

Not applicable.
